# Reaching within a dynamic virtual environment

**DOI:** 10.1186/1743-0003-4-23

**Published:** 2007-07-04

**Authors:** Assaf Y Dvorkin, Robert V Kenyon, Emily A Keshner

**Affiliations:** 1 Sensory Motor Performance Program, Rehabilitation Institute of Chicago, 345 East Superior Street, Chicago, IL, 60611, USA; 2 Department of Computer Science, University of Illinois at Chicago, Chicago, IL, USA; 3 Department of Physical Medicine and Rehabilitation, Feinberg School of Medicine, Northwestern University, Chicago, IL, USA; 4 Department of Physical Therapy, College of Health Professions, Temple University, Jones 600, 3307 Broad St, Philadelphia, 9140, USA

## Abstract

**Background:**

Planning and execution of reaching requires a series of computational processes that involve localization of both the target and initial arm position, and the translation of this spatial information into appropriate motor commands that bring the hand to the target. We have investigated the effects of shifting the visual field on visuomotor control using a virtual visual environment in order to determine how changes in visuo-spatial relations alter motor planning during a reach.

**Methods:**

Five healthy subjects were seated in front of an immersive, stereo virtual scene while reaching for a visual target that remained stationary in space or unpredictably shifted to a second position (either to the right or left of the first target) with different inter-stimulus intervals. Motion of the scene either matched the motion of their head or was rotated counter clockwise at 130 deg/s in the roll plane.

**Results:**

Initial results suggested that both the temporal and spatial aspects of reaching were affected by a rolling visual field. Subjects were able to amend ongoing motion to match target position regardless of scene motion, but the presence of visual field motion produced significantly longer pauses during the reach movement when the target was shifted in space. In addition, terminal arm posture exhibited a drift in the direction opposite to the roll motion.

**Conclusion:**

These findings suggest that roll motion of the visual field of view interfered with the ability to imultaneously process two consecutive stimuli. Observed changes in arm position following the termination of the reach suggest that subjects were compensating for a perceived change in their visual reference frame.

## Background

During the execution of a motor task, the central nervous system (CNS) monitors online body orientation by updating the internal representation of visual space. Studies have shown that both young and elderly healthy subjects are able to amend their ongoing movements in response to target displacement during a "double-step" paradigm which changes the spatial goal of the movement by unexpectedly changing the location of a visual target [1-4]. However, these movements have only been tested in stationary visual environments. During most active motions the individual and the external world are moving at the same time. While there is ample evidence that dynamic visual inputs affect motor behavior, (e.g., disrupting upper extremity movement trajectory and endpoint [5] and increasing postural instability [6,7]), the weighting of such visual information and the exact role that visual motion plays in human motor control is not well understood. In recent years, virtual reality technology has emerged as a powerful tool to study motor control in healthy subjects and in patients with stroke or labyrinthine deficiency [8-10] because it enables us to manipulate the visual world. In the current study we examined how motion of a virtual environment (VE) might affect planning and execution of three-dimensional (3D) reaching movements using the double-step paradigm. We hypothesized that roll motion of the visual field, which was found to produce robust postural changes [7], would affect timing and position of the arm in space. Furthermore, we hypothesized that reaching toward a remembered target location would enhance this effect of visual field motion on performance.

## Materials and Methods

### Subjects

Five young healthy adults (age 25–35 years) participated in the study. All subjects were right-handed and had normal or corrected-to-normal vision. Subjects gave informed consent in accordance with the Institutional Review Board of Northwestern University.

### Apparatus and data collection

**Figure 1:**
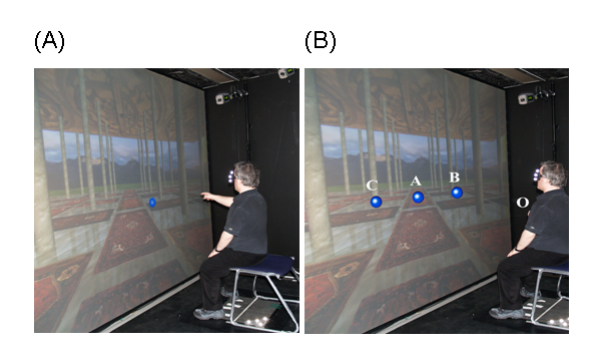
(A) Screen shot of an individual performing within the VE. (B) Spatial arrangement of the visual targets ('A', 'B' and 'C'), and initial hand position ('O') which was located on the sternum. Target positions were defined in terms of the subject's arm length and sternum position. Note that the letter labels do not appear within the VE.

The VE and the hardware and software responsible for its generation have been previously reported [6]. In brief, subjects were exposed to an immersive 3D wide field of view VE (scene), projected onto a 2.6 m × 3.2 m back-projection screen. Visual targets, which appeared with the scene, were generated as 3D virtual ball-shaped targets with a 1 cm radius (Figure 1a and 1b). Current orientation of the stereo shutter glasses, worn by the subject (Crystal Eyes, StereoGraphics Inc.), determined the correct perspective for the scene. Hand 3D movements were recorded using a six camera Motion Analysis system (Motion Analysis, Inc.). Reflective markers attached to the right arm, head, and trunk, were tracked at 120 Hz.

### Procedures

Subjects sat 1.2 m from the screen for two experimental protocols that controlled sequence and duration of the targets' appearance. In the first experiment, five blocks of trials were presented, each containing 12 single-step and 24 double-step trials in random order. The visual scene was either matched to motion of the head, or it rotated counter clockwise about the line of sight (rolling scene) at a constant velocity of 130 deg/s. In a single-step trial, a visual target appeared for 2 s. In a double-step trial, the central target appeared. Following a pre-specified inter-stimulus interval (ISI) of 50, 200 or 500 ms (either before or following movement initiation), the location of that target shifted either left or right and remained in the new position for 2 s. The scene either remained matched to head motion or started to roll as soon as a target appeared within the scene. Subjects were instructed to reach toward the target as soon as it appeared, and to move the hand towards the new location if the target changed position, (head and trunk were free to move). Subjects were also instructed to keep their hand at the final position until the scene turned black which signaled the end of the trial.

To investigate whether reaching toward a remembered target location enhanced the effect of roll motion on performance, a second experiment tested changes in the duration of target appearance. The target in the single-step condition and the final target in the double-step condition were visible for only 200 ms. ISI values were 200 and 500 ms. Each block contained a mixture of 12 single-step and 16 double-step trials.

### Data analysis

Data from the measured hand position were low-pass filtered off-line at 8 Hz using a 4^th ^order Butterworth digital filter. A 4% peak velocity threshold determined movement onset and offset. Hand path and the proportion and duration of pauses that occurred during the course of the movement (an interval of at least 40 ms in which the hand was stationary) were calculated from hand position and velocity.

## Results

**Figure 2:**
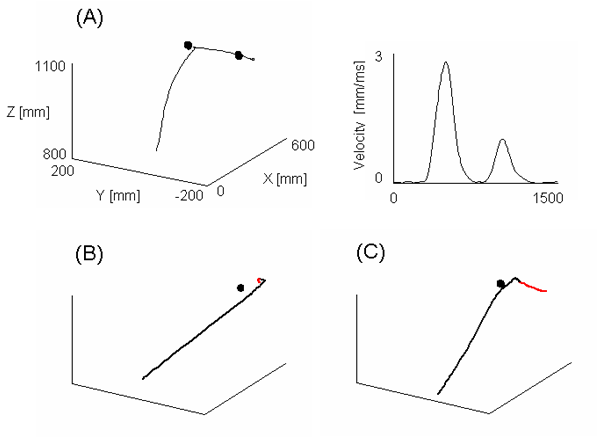
(A) Examples of 3D path and the corresponding tangential velocity profile of a paused movement (subject paused for 180 ms). (B) and (C) Examples of representative single-step 3D paths showing a stable final hand position and an additional movement of the hand (in red) following the main movement (in black), respectively. Targets appear as black circles.

Kinematics of the reaching motion within the VE was characterized by similar properties to those described in previous studies of reaching in the physical world, e.g., [4]. Subjects were able to amend their ongoing motion in response to target displacement in both experiments and with both scene conditions. They exhibited single- and double-peaked bell-shaped velocity profiles for the single- and double-step conditions, respectively. In addition, some double-step movements exhibited a pause prior to modifying the arm trajectory (Figure 2a). The proportion of paused movements in the total double-step movements was lower for Experiment 1 (15.7%) than Experiment 2 (27.7%). In addition, mean duration of paused movement was significantly shorter in Experiment 1 than Experiment 2 (117 vs. 156 ms; χ^2 ^_(1) _= 5.57, p = 0.018). Although the proportion of paused movements was similar with both scene conditions in both experiments, the duration of the pause was significantly different between the scene that was matched to head motion (120 ms) and the rolling scene (190 ms) in Experiment 2 (χ^2 ^_(1) _= 15.8, p < 0.0001). Furthermore, the 3D hand path was consistently curved for both single- and double-step conditions, irrespective of target position and scene condition. Overshoots but especially undershoots with respect to the subject's body were observed across all subjects.

Finally, no obvious changes in the trunk and head position were observed during both experiments. In addition, all subjects were able to maintain the final hand position for both scene conditions in Experiment 1 (Figure 2b). Following the main reaching movement with a rolling scene in Experiment 2, however, the hand continuously moved slowly toward the right which was opposite the direction of the rolling scene (Figure 2c).

## Discussion

An earlier study [3] which used the double-step task in a stationary VE demonstrated that subjects modified hand trajectory in response to target displacement. We have now shown that this holds true with a moving virtual scene suggesting that our results will transfer to the physical world. Both the temporal and spatial aspects of the reach movement were affected by roll motion of the visual scene. Reach was affected both during and following the movement towards the target. Mean pause duration during the course of the reaching movement increased with roll of the visual scene, implying that visual motion interfered with the ability to simultaneously prepare motor responses to the two consecutive visual targets [4,11]. Following termination of the reach, a drift in hand position was observed only during roll motion and in the absence of a target (Experiment 2). We infer that subjects were compensating for motion of the visual surround which produced a perceived change in their visual reference frame.

No postural changes were observed in our data even though a conflict existed between the signals from the visual, vestibular, and somatosensory systems [6]. The absence of body tilt suggests that support surface inputs from the seat were more heavily weighted than the sensory conflict arising from roll motion of the visual field.

## Conclusion

These initial results demonstrate that motion of the visual field affected both planning and execution of the reaching movement, particularly while reaching toward a remembered target. Reaching within a moving visual environment involves complex sensorimotor transformations as a result of the continuous change in the visual reference frame which could be used to promote adaptation and learning in individuals with CNS injury. Thus these data could eventually lead to rehabilitation paradigms that minimize the disturbing effect of visual motion on motor planning and execution. Knowledge of how visual motion affects both reaching and postural control might have ramifications for both elderly subjects and labyrinthine deficient individuals who have been shown to experience post-rotation disturbances of posture, gait, and arm movement control [12].

## Competing interests

The author(s) declare that they have no competing interests.

## Authors' contributions

AYD designed and conducted the experiment, performed the analysis and wrote the manuscript. RVK participated in the design of the study and was involved in revising the manuscript. EAK participated in the design of the study, made substantial contribution for the interpretation of data and was involved in revising the manuscript. All authors read and approved the final manuscript.
